# Animal Chemistry

**Published:** 1838-01

**Authors:** 


### ANIMAL CHEMISTRY.

### *Microscopical Observations relative to Stains upon Linen.* By M. Rattier.

The most simple method for this mode of investigation consists in separating, if possible, the part on which the experiment is to be made, placing it in a vessel of moderate depth (such as a watch-glass,) and pouring upon it some distilled water to the depth of one or two lines. As soon as the stain is well soaked, its surface is to be slightly scratched with a spatula; in some cases, where the matter is in very great quantity, it is sufficient to shake the liquid for a moment. If we then submit the solution thus obtained to the microscope, we shall find, if the stain is from blood, globules of blood, some of which are deformed, in proportion to the antiquity of the stain and the rubbing it has sustained; but, in the majority of cases, they will be always found presenting such characters that, if they are once seen, cannot be mistaken. In the solution of mucus, of pus, and of semen, especially of pus, the globules are seen with the greatest facility; and, in the solution of semen, in the midst of the remains of spermatic animalcules, we shall always find some entire.

*Journal de Chimie Médicale. March,* 1837.

---

### *Observations on Blue Urine.* By M. Drantz.

Of all the changes of which the urine is susceptible, none are more remarkable than the various colours it assumes in different diseases, and of the nature of which we know so little. M. Braconnot, in analyzing some blue urine, obtained, by simple filtration, a matter of the same colour, to which he has given the name of cyanourine. MM. Julia de Fontenelle, Mojon, and Cantu have announced that they have ascertained that this colour was due to hydroferrocyanate of iron (Prussian blue). M. Drantz took the opportunity of examining a small quantity of blue urine passed by a young man afflicted with influenza. It was of a deep blue colour, deposited, upon standing, a blue matter, which had all the properties of hydroferrocyanate of iron. Deprived of the colouring matter by means of filtration, it gave, upon analyzation, a large quantity of albumen, gelatin, salts and some traces of urea. The patient was not making use of any ferruginous preparation at the time. *Journal de Chimie Médicale. June,* 1837.

